# Evaluation of Sampling Recommendations From the Influenza Virologic Surveillance Right Size Roadmap for Idaho

**DOI:** 10.2196/publichealth.6648

**Published:** 2017-08-24

**Authors:** Mariana Rosenthal, Katey Anderson, Leslie Tengelsen, Kris Carter, Christine Hahn, Christopher Ball

**Affiliations:** ^1^ Centers for Disease Control and Prevention Idaho Department of Health and Welfare Boise, ID United States; ^2^ Idaho Bureau of Laboratories Idaho Department of Health and Welfare Boise, ID United States; ^3^ Division of Public Health Idaho Department of Health and Welfare Boise, ID United States

**Keywords:** influenza, sample size, public health surveillance

## Abstract

**Background:**

The Right Size Roadmap was developed by the Association of Public Health Laboratories and the Centers for Disease Control and Prevention to improve influenza virologic surveillance efficiency. Guidelines were provided to state health departments regarding representativeness and statistical estimates of specimen numbers needed for seasonal influenza situational awareness, rare or novel influenza virus detection, and rare or novel influenza virus investigation.

**Objective:**

The aim of this study was to compare Roadmap sampling recommendations with Idaho’s influenza virologic surveillance to determine implementation feasibility.

**Methods:**

We calculated the proportion of medically attended influenza-like illness (MA-ILI) from Idaho’s influenza-like illness surveillance among outpatients during October 2008 to May 2014, applied data to Roadmap-provided sample size calculators, and compared calculations with actual numbers of specimens tested for influenza by the Idaho Bureau of Laboratories (IBL). We assessed representativeness among patients’ tested specimens to census estimates by age, sex, and health district residence.

**Results:**

Among outpatients surveilled, Idaho’s mean annual proportion of MA-ILI was 2.30% (20,834/905,818) during a 5-year period. Thus, according to Roadmap recommendations, Idaho needs to collect 128 specimens from MA-ILI patients/week for situational awareness, 1496 influenza-positive specimens/week for detection of a rare or novel influenza virus at 0.2% prevalence, and after detection, 478 specimens/week to confirm true prevalence is ≤2% of influenza-positive samples. The mean number of respiratory specimens Idaho tested for influenza/week, excluding the 2009-2010 influenza season, ranged from 6 to 24. Various influenza virus types and subtypes were collected and specimen submission sources were representative in terms of geographic distribution, patient age range and sex, and disease severity.

**Conclusions:**

Insufficient numbers of respiratory specimens are submitted to IBL for influenza laboratory testing. Increased specimen submission would facilitate meeting Roadmap sample size recommendations.

## Introduction

Influenza illness in the United States produces a significant burden in terms of morbidity, mortality, and economic influence. An average of >200,000 influenza-associated hospitalizations and >23,600 influenza-associated deaths with underlying respiratory and circulatory causes have been estimated to occur in the United States annually [1,2]. The total economic burden of annual influenza epidemics by using projected statistical life values has been estimated at US $87.1 billion [3]. Whereas hospitalization costs are important contributors, lost productivity from missed work and lost lives comprise the bulk of the economic burden. During October 2008 to May 2014 in Idaho, according to Influenza-like Illness National Surveillance Network (ILINet), the mean annual proportion of outpatient visits for influenza-like illness (ILI) was 20,834 (2.3%) out of 905,818 total outpatient visits. ILINet is a national network maintained by the Centers for Disease Control and Prevention (CDC) that consists of more than 2800 enrolled outpatient health care providers reporting patient visits due to ILI. The Idaho Division of Public Health (DPH), Bureau of Vital Records and Health Statistics reports that 103 influenza and influenza-related deaths occurred in Idaho during this period.

National influenza virologic surveillance is conducted by 60 National Respiratory and Enteric Virus Surveillance System (NREVSS) laboratories and approximately 85 US World Health Organization (WHO) collaborating laboratories located throughout the United States [4]. The DPH Idaho Bureau of Laboratories (IBL) participates as a US WHO collaborating laboratory. IBL conducts influenza virologic surveillance to identify circulating strains, identify antiviral resistance, and detect novel strains in Idaho. Novel influenza is reportable in Idaho, but seasonal influenza is not reportable except during outbreak settings, therefore specimen submission in support of seasonal surveillance is voluntary and is based on convenience sampling. IBL engages ILINet sites and other health care providers and laboratories around the state, including hospital laboratories, to voluntarily submit respiratory specimens from those with medically attended ILI (MA-ILI), prescreened for influenza or not, for reverse-transcription polymerase chain reaction testing for influenza A and B, and subtypes AH1, AH1N1, AH3, and AH5. Isolates, including untypeable isolates, are sent to the CDC for genotyping. Specimen collection is year-round; however, solicitation efforts are heightened during influenza season, which runs from *Morbidity and Mortality Weekly Report* (*MMWR*) epidemiologic week 40 through *MMWR* epidemiologic week 20, typically from the beginning of October through mid-May.

During 2013, the Association of Public Health Laboratories and CDC developed the Influenza Virologic Surveillance Right Size Roadmap (Roadmap) [5]. The Roadmap is a resource to assist state health departments in optimizing virologic surveillance system by helping to identify the number of specimens to be tested to ensure ample confidence in influenza surveillance and detection of novel viruses. Sampling recommendations are included with the Roadmap for improved influenza virologic surveillance. Sample size calculators [6] were developed to systematically establish virologic sample size goals on the basis of minimum detection thresholds and acceptable confidence levels for 3 different surveillance objectives as follows: (1) situational awareness—to determine the beginning and end of the influenza season and monitor the prevalence and spread of influenza viruses throughout the year; (2) rare or novel influenza event detection—to detect a rare or novel influenza virus among influenza-positive specimens tested in the United States at a low enough threshold for effective intervention and control measures; and (3) rare or novel influenza event investigation—to determine the prevalence of the rare or novel influenza virus and confirm it does not exceed a specific percent positivity, within a state (eg, Idaho) after the initial detection of a rare or novel influenza virus. Components of the sampling recommendations include using “a statistical, systematic approach to collect an appropriate, adequate number of specimens” representative of virus type and subtype, entire year, geographic and age diversity of the population, and influenza disease severity [5]. Moreover, the Roadmap recommends ensuring a timely information flow, through the 5 tiers of influenza virus surveillance from point-of-care settings to CDC’s laboratories. We compared the Roadmap sampling recommendations with Idaho’s influenza virologic surveillance to determine implementation feasibility. That is, the comparison was made to see whether any gaps between them might help determine whether it would be feasible for Idaho’s influenza virologic surveillance to meet the recommended sampling goals.

## Methods

We included the previous 5 years’ worth of available data to capture any unusual fluctuations in sample collection, as shown in 2009-2010 due to the H1N1 pandemic. Additionally, 5 years of data allow for the possibility of noting sample collection trends. We calculated MA-ILI proportion by using data from Idaho’s outpatient ILI surveillance during October 2008 to May 2014. Data regarding outpatient visits for ILI were collected through CDC’s ILINet, which defines ILI as fever (≥100°F) and a cough or sore throat without a known cause other than influenza. Eleven health care sites from Idaho participated in this surveillance by providing weekly the total number of patients who sought outpatient care specifically for ILI and the number of patients treated for any reason. We used the latest available estimates from the US Census Bureau for Idaho [7] to determine population age and sex distribution in the state. We obtained the total number of respiratory specimens tested for influenza at IBL during October 2008 to May 2014 from the influenza virologic surveillance system in Idaho.

We used the calculated proportion of baseline MA-ILI and the preestablished estimated Idaho population size as a starting point in the 3 Roadmap sample size calculators, and compared these calculations with actual numbers of specimens tested for influenza by IBL. We assessed representativeness of the tested specimens in terms of submission month and year, virus type and subtype, patient’s county of residence and health district, patient age and sex, and patient hospitalization status. We compared the proportion of females and the proportion of patients aged <5 years or >65 years among tested specimens with the proportion of females and the proportion of persons aged <5 years or >65 years from Idaho residents, respectively. We assessed flow of specimens in terms of timeliness and determined whether they were prescreened at the health care-provider level before submission. Calculations were done by using Microsoft Excel 2010 (Microsoft Corporation, Redmond, Washington).

This study underwent CDC human subjects review and was deemed not to involve human subject research.

## Results

According to ILINet, the mean baseline annual proportion of MA-ILI in Idaho during October 2008 to May 2014 was 2.3%. At this proportion, for influenza situational awareness in Idaho, IBL would need test results for 128 specimens from MA-ILI patients/week to determine that the prevalence of influenza-positive specimens is 10% (considered the start of the influenza season; seasonal baseline setting recommended by the Roadmap, page 58) at a 95% confidence level and 5% error rate (situational awareness setting recommended by the Roadmap, page 52) (Table 1) [5]. At the peak of influenza seasons, based on an annual proportion of MA-ILI of 5%, IBL would need to test results for 297 specimens from MA-ILI patients/week to determine that the prevalence of influenza-positive specimens is 30% at a 95% confidence level and 5% error rate. For rare or novel influenza event detection, IBL would need test results from 1496 influenza-positive specimens/week to allow the Idaho surveillance system to detect a rare or novel influenza virus at 0.2% prevalence at a 95% confidence level (Table 1). For rare or novel influenza event investigation after a rare or novel influenza virus is detected, IBL would need test results from 478 MA-ILI specimens/week to confirm that the true prevalence does not exceed 2% of influenza-positive within the state at a 95% confidence level (Table 1). If surveillance is done at the national rather than state level, the minimum number of influenza-positive specimens that Idaho would need to meet the second 2 surveillance objectives decreases from 1496 to 8 for detection of a rare or novel influenza event nationally at a 0.2% threshold, and from 478 to 3 for a 95% confidence level that the actual national prevalence of the novel virus does not exceed 2% of the influenza-positive specimens (Table 1).

**Figure 1:**
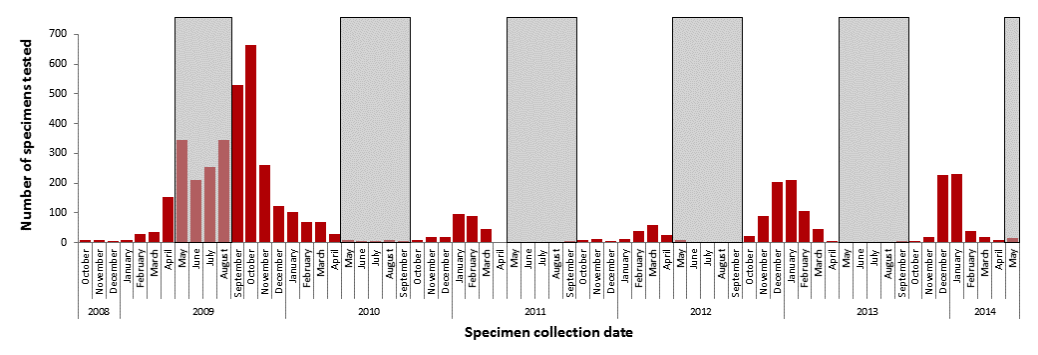
Number of influenza specimens tested at Idaho Bureau of Laboratories by month, October 2008 to May 2014 (N=4984). Shading denotes months outside of traditional influenza seasons. Seasonal influenza activity is illustrated by the sinusoidal shape of the bar graph.

During October 2008 to May 2014, 4984 respiratory specimens were tested for influenza at IBL. In 5 of 6 influenza seasons included in the study period, the pattern of specimen submission coincided with typical influenza seasons; during the 2009-2010 influenza A (H1N1) pandemic there was an uncharacteristic increase in specimen submission (Figure 1). The average number of respiratory specimens tested for influenza/week, excluding the 2009-2010 influenza season, ranged from 6 to 24 (Table 2). During the 2009-2010 pandemic influenza season, this increased to 47 samples/week.

IBL received specimens during 64 (94%) of 68 months evaluated; however, during 31 (46%) months, ≤10 samples were submitted. Sample test results represented nationally circulating virus types and subtypes, including influenza A (H1 and H3) and influenza B. Specimen submission sources were geographically diverse, with 37 of 44 counties of residence and all 7 public health districts *represented*. Patient age ranged from 1 to 101 years. Persons aged <5 years or >65 years comprised 21.5% of the state population [7], and 23.9% of patients from whom specimens were submitted. Females comprised 49.9% of the Idaho population according to the 2015 US Census Bureau estimates [7], and 53.5% of patients from whom specimens were submitted. Hospitalization status of patients was used to assess representativeness regarding disease severity and was available for 89.2% of specimens: 47.9% and 41.3% of tested specimens came from hospitalized and nonhospitalized patients, respectively.

**Table 1:** Recommended number per week of respiratory specimens to be tested for influenza per Roadmap surveillance objective in Idaho.

Objective	Number of recommended specimens to sample per week
Objective 1^a^	128
Objective 2^b^	1496 (8 if National surveillance)
Objective 3^c^	478 (3 if National surveillance)

^a^To determine that the prevalence of influenza-positive specimens is 10% at a 95% confidence level and 5% error rate.

^b^To allow the Idaho surveillance system to detect a rare or novel influenza virus at 0.2% prevalence at a 95% confidence level.

^c^To confirm that the true prevalence does not exceed 2% of influenza-positive within the state at a 95% confidence level.

**Table 2:** Total number and average per week of respiratory specimens tested for influenza per influenza season at the Idaho Bureau of Laboratories, by influenza season in Idaho, 2008-2014.

Year of influenza season (October 1 to May 1)	2008-2009	2009-2010	2010-2011	2011-2012	2012-2013	2013-2014
Total number of specimens tested	243	1317	278	160	677	545
Average number of specimens/week	9	47	10	6	24	20

The flow of respiratory specimens seemed timely. CDC recommends that influenza specimens should be submitted as they are collected and not batched, and be tested within 72 hours of collection for optimal virus recovery [8,9]. IBL received specimens an average of 2.4 days after collection, tested 80.6% within the first week postreceipt, and uploaded results into the Laboratory Information Managing System immediately. Test result summaries were sent to CDC weekly. Approximately 97% of respiratory specimens were prescreened for influenza at the health care-provider level before IBL submission, with the majority (85.8%) being influenza rapid test-positive.

## Discussion

Although Idaho’s virologic surveillance system fails to meet all Roadmap sampling recommendations for chosen parameters used in this study, DPH has the ability to meet the sample size recommendation for situational awareness if it were able to supplement data from IBL, a public health laboratory, with virologic surveillance data from other sources, such as clinical or commercial laboratories, including participating Idaho NREVSS sites or other points of care in Idaho as recommended by the Roadmap to supplement (Roadmap, page 78). Additionally, IBL alone would be able to meet sample size recommendations if testing specimens received in formats other than in viral transport media were approved for diagnostic use. CDC’s interim guidance on testing, collecting, and processing specimens for influenza surveillance indicates that the preferred respiratory specimens for submission should be placed into sterile viral transport media and immediately placed on refrigerant gel-packs or at 4°C for transport [8,9]. Unfortunately, submission of leftover clinical samples such as those from a rapid test done at a clinical setting is not approved or recommended as an optimal specimen for diagnostic testing. However, there is evidence that influenza infection cannot be ruled out by negative rapid test results due to potential low sensitivity of the diagnostic test; researchers have successfully been able to quantify influenza viral loads from false-negative viral suspensions left over from influenza rapid tests [10]. For that reason, we envisaged this specimen type as a suitable alternative for virologic influenza surveillance testing and suggest it as one way to augment sample submission numbers. Moreover, if surveillance is done at the national rather than state level, number of specimens recommended by the Roadmap to be tested from Idaho for both detection and investigation of a rare or novel influenza event decreases substantially. However, system sensitivity to detect and investigate a rare or novel influenza event at the national level relies on the contribution of all states to submit proportionate numbers of specimens and data to their population size.

Roadmap calculators are subjected to arbitrarily chosen parameters, including influenza positivity, confidence levels, error rates, and detection thresholds. This flexibility allows states to adjust their target number of influenza virologic specimens to be tested to the realities of sample collection and time frame during the influenza season or outbreak. While selecting higher margins of error allows for a wider tolerance for error in sampling recommendations, in a practical situation, a surveillance system might find that meeting such relaxed sampling sizes could result in miscalculating the start of influenza season or the detection of a rare or novel influenza event. Convenient, precalculated tables for situational awareness and for rare or novel influenza event detection are available in the Roadmap Appendix B for quick reference, and offer the user a range of arbitrarily chosen parameters to determine the variability of recommended target sample sizes based on a few different population sizes. Using the Roadmap calculators with user-specified inputs provides an increased precision and allows the user to understand the weight of each parameter by visually noting the effect of altering each during calculations. Calculators also allow the user to work backwards by inputting actual sample sizes obtained by their state and determining confidence levels regarding the true influenza prevalence in their state. No precalculated tables for rare or novel influenza event investigation are available.

During October 2008 to May 2014 in Idaho, respiratory specimens collected were representative in terms of virus type and subtype (eg, influenza A [H1 and H3] and influenza B), time (eg, 94% of the 68 months evaluated), geography (eg, all of Idaho’s public health districts, including 37/44 Idaho counties), age diversity of the population (eg, 1-101 years), and influenza disease severity, measured by hospitalization status (eg, 47.9% hospitalized versus 41.3% nonhospitalized). Respiratory specimens were also processed within a timely manner, ensuring a timely flow from the patient level to the CDC level of virologic surveillance. A substantial portion of specimens submitted to IBL were initially screened positive with a rapid test for influenza by submitters, likely altering the positivity rate and biasing prevalence calculations upward. Although realistically difficult to do under a resource-limited, voluntary submission system, submitters should be encouraged to submit specimens from both influenza-positive and -negative tests when possible.

During October 2008 to May 2014, the number of respiratory samples submitted to IBL for influenza testing was below Roadmap recommendations. Implementing an incentive program to increase specimen submission might be beneficial. Additional incentives for further collaboration among health care entities and state public health laboratories would be helpful. Agreements with hospitals for sharing data from implemented multiplex respiratory panels that include influenza types and subtypes might be helpful in determining the proportion of influenza-positive and influenza-negative specimens from the total tested, and in meeting Roadmap surveillance objectives.

States can benefit from using the Web-based sample size tools [6] provided in the Roadmap, because of the ease with which they can determine their capacity to attain situational awareness and detect and investigate the occurrence of a rare or novel influenza strain. Moreover, states can assess how their contributions add to the collaborative effort needed to perform national influenza virologic surveillance.

## Abbreviations

CDC: Centers for Disease Control and Prevention
DPH: Division of Public Health
IBL: Idaho Bureau of Laboratories
ILI: influenza-like illness
ILINet: Influenza-like Illness National Surveillance Network
MA-ILI: medically attended influenza-like illness
MMWR: Morbidity and Mortality Weekly Report
NREVSS: National Respiratory and Enteric Virus Surveillance System
WHO: World Health Organization
